# The reservoir of latent HIV

**DOI:** 10.3389/fcimb.2022.945956

**Published:** 2022-07-28

**Authors:** Jing Chen, Tong Zhou, Yuan Zhang, Shumin Luo, Huan Chen, Dexi Chen, Chuanyun Li, Weihua Li

**Affiliations:** ^1^ Beijing Institute of Hepatology, Beijing Youan Hospital, Capital Medical University, Beijing, China; ^2^ Xiangya School of Medicine, Central South University, Changsha, China; ^3^ Beijing Youan Hospital, Capital Medical University, Beijing, China

**Keywords:** reservoir of latent HIV, formation, cell reservoir, tissue reservoir, detection, remove

## Abstract

The persistence of latent reservoir of the human immunodeficiency virus (HIV) is currently the major challenge in curing HIV infection. After HIV infects the human body, the latent HIV is unable to be recognized by the body’s immune system. Currently, the widely adopted antiretroviral therapy (ART) is also unble to eliminate it, thus hindering the progress of HIV treatment. This review discusses the existence of latent HIV vault for HIV treatment, its formation and factors affecting its formation, cell, and tissue localization, methods for detection and removing latent reservoir, to provide a comprehensive understanding of latent HIV vault, in order to assist in the future research and play a potential role in achieving HIV treatment.

## Introduction

HIV is a global health problem. At present, nearly 37 million people around the world are living with HIV infection (Ortblad et al., 2013). HIV evades the host immunity in many ways after infection (Theys et al., 2018; Beitari et al., 2019; Yin et al., 2020), among which the most common is through the establishment of latent reservoir of the HIV (Sengupta and Siliciano, 2018; Pedro et al., 2019). After HIV infects activated CD4^+^T lymphocytes, it produces a large number of viral RNA and viral proteins, which are eventually recognized and killed by the host immune system (van Zyl et al., 2018). Subsequently, some HIV after integration into the host cells mainly exist in CD4^+^T cells of resting memory, and constitute the latent reservoir of HIV (Bruner et al., 2019). These cells carry the integrated latent protovirus and exist through homeostasis or antigen-driven proliferation (Shan et al., 2017; Rezaei et al., 2018). At present, ART control the HIV level in people living with HIV (PWLH) below detection line, which plays an important role in the process of antiviral therapy (Lu et al., 2018). However, due to the existence of a latent HIV reservoir, ART cannot eliminate the latent virus in the reservoir, and HIV will rebound once the treatment is stopped (Xiao et al., 2019; Cohn et al., 2020). HIV cannot be completely cured, although some breakthroughs have been made in the research field of latent HIV reservoir. However, its specific mechanism of action is not completely clear. This review provides insight into the formation, location, detection and elimination of latent HIV reservoir.

## Formation of latent HIV reservoir and its influencing factors

**Figure 1:**
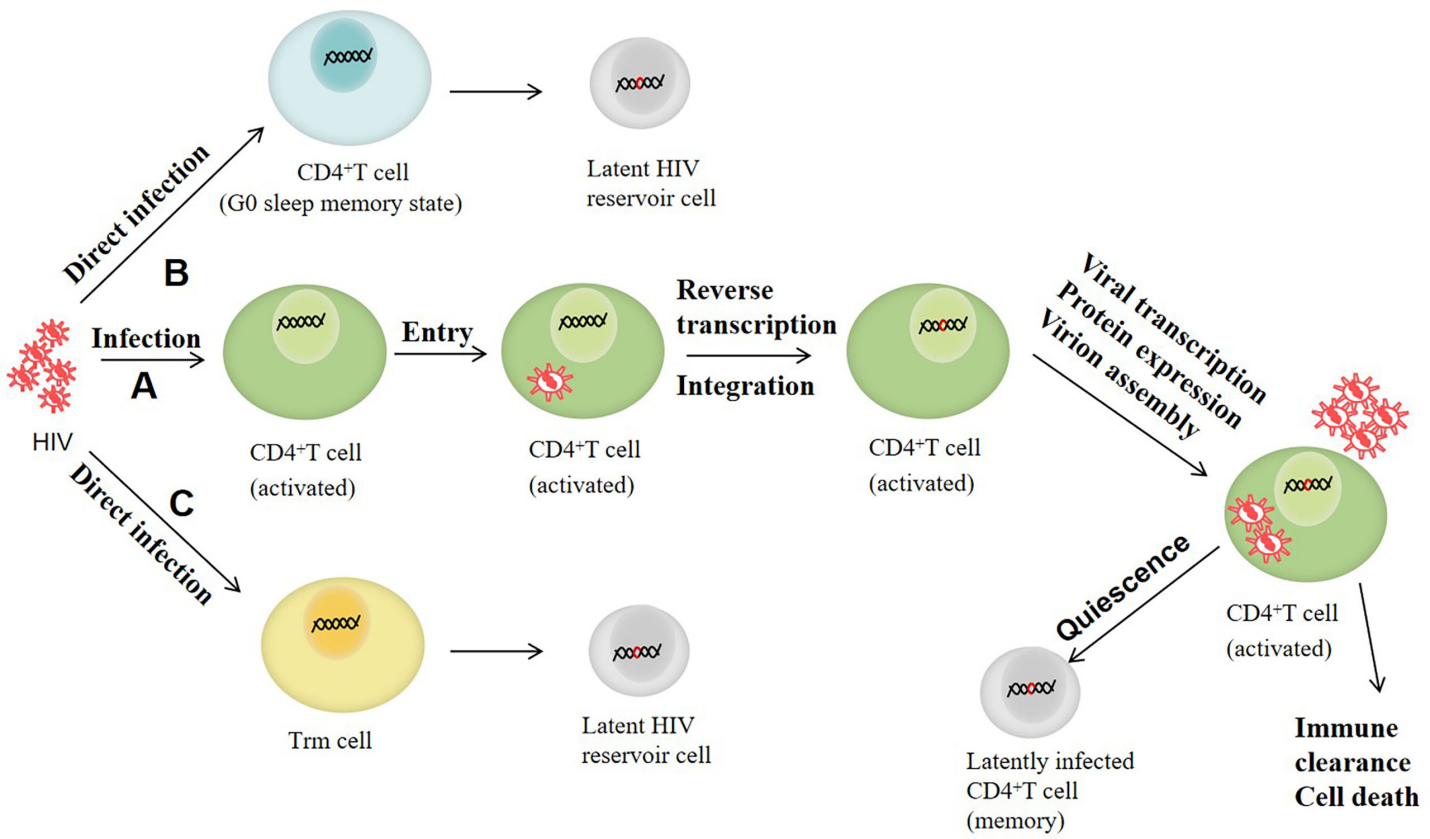
The formation process of latent HIV reservoir. **(A)** HIV mainly infects human CD4+T lymphocytes. When it enters the cell, the RNA is first reversely transcribed into HIV DNA, which is then incorporated into the DNA of CD4+T cells. Some CD4+T cells with integrated HIV DNA are converted into a resting state, and the HIV in the resting CD4+T becomes latent HIV. **(B)** HIV directly infects CD4+T cells that revert to a G0 dormant memory state, thus enabling the virus to enter latency. **(C)** HIV establishes incubation by directly infecting resting memory CD4+T cells (Trm cells).

The reservoir of latent HIV can be established in the early stage of infection and is mainly composed of CD4^+^T cells with resting memory (Moranguinho and Valente, 2020). After HIV enters the human body, it mainly infects human CD4^+^T lymphocytes. The RNA is first reversely transcribed into HIV DNA, which is then integrated into the DNA of CD4^+^T cells, part of which transforms into a resting state in oder to inhibit the viral gene expression. The HIV in these resting CD4^+^T cells becomes the latent HIV, which is the main formation mode of the latent HIV reservoir at present (García et al., 2020). In addition, with the advent of research, other mechanisms for the establishment of latent HIV reservoirs have also been proposed. For instance, HIV can directly infect CD4^+^T cells that revert to a G0 dormant memory state, thus enabling the virus to enter latency (García et al., 2018). It has been suggested that latency may be established by direct infection of resting memory CD4^+^T cells (Trm cells) (García et al., 2020; O’Neil et al., 2021). Selective reverse transcriptional products tyrosine aminotransferase (Tat) and negative factor (Nef) exist in Trm cells (Schulze-Gahmen and Hurley, 2018), and can induce cell activation so that the virus genome can be integrated into the cell genome (Donahue et al., 2013; Pinto et al., 2020). Moreover, it has also been found that although Trm cells are resistant to HIV compared with activated CD4^+^T cells, mild stimulation of chemokine CC ligand 19 (CCL19) and chemokine CC ligand 21 (CCL21) and cytokines interleukin (IL)-4 and interleukin (IL)-7 can promote the direct infection of resting CD4^+^T cells with HIV without inducing significant T cell activation (Saleh et al., 2007; Wu, 2010; López-Huertas et al., 2019). The establishment and maintenance of latent infection may be influenced by a variety of factors, including the availability of host transcription factors, epigenetic modifications, HIV Tat protein defects, integration sites and directions, and post-transcriptional regulatory mechanisms. After the reservoir of latent HIV is established, the transcription level of HIV in the reservoir is very low, and almost no virus is produced as there is no viral protein, and the latent infected cells will not be affected by cytotoxicity, nor will they be recognized by the immune system, so that they can exist stably for a long time. **Figure 1** shows the main formation process of latent HIV reservoir.

The factors affecting the formation of latent HIV reservoir can be generally divided into two aspects: inhibiting the formation of latent HIV reservoir and promoting the formation of latent HIV reservoir. B-cell lymphoma 2 (BCL2) is a key regulatory molecule of lymphoid tissue homeostasis, affecting HIV homeostasis in infected lymphocytes. BCL2 can directly bind Casp8p41 and prevent the latter from binding and activating Bcl-2 homolog antagonist/killer (BAK), thereby inducing apoptosis (Cummins et al., 2016). It has been found that the expression of Casp8p41 in resting memory CD4^+^T cells is negatively correlated with the absolute CD4^+^T count. BCL2 can inhibit the formation of latent HIV reservoirs depending on the enhanced cytotoxicity of Casp8p41 (Cummins et al., 2017). In addition, the purified Tat protein inhibits the establishment of latent HIV reservoir without changing the susceptibility of cells to HIV (Donahue et al., 2012).

On the contrary, some substances may also promote the formation of latent HIV reservoirs. Programmed cell death protein 1 (PD-1) and lymphocyte activation gene 3 (LAG-3) were initially identified as markers of HIV-infected cells (Fromentin et al., 2016). PD-1 plays an active role in silencing HIV transcription (Boussiotis and Patsoukis, 2022). A recent study reported that the TRIM28 protein helps in binding a chemical marker called SUMOylation to the cell’s gene regulators and inhibits the activity of HIV genes, thereby inhibiting its expression and promoting latency (Ma et al., 2019). The binding of interferon-γ-induced protein (IP) -10 to C-X-C chemokine receptor 3 (CXCR3) has also been reported to enhance latent infection of resting CD4^+^T cells against HIV (Wang et al., 2021). IP-10 stimulation promotes cofilin activity and actin dynamics, thereby promoting HIV entry and DNA integration, suggesting that IP-10 is also a key factor in the formation of latent HIV reservoir, and targeting IP-10 therapy may be a potential strategy in inhibiting latent HIV infection (Lei et al., 2019; Wang et al., 2021). In addition, cell-to-cell contact between infected cells and uninfected cells is also a key feature in the formation of an HIV latent virus reservoir (Pedro et al., 2019; Okutomi et al., 2020). Cell-to-cell contact enhances the susceptibility of resting CD4^+^T cells to HIV (Agosto et al., 2018). Previous studies have shown that monocytes or myeloid dendritic cells (mDCs) co-culturing with activated HIV-infected T cells may facilitate their transition to the activated latent state, highlighting the role of intercellular contact in the establishment of HIV latent reservoir. Furthermore, cytokines are also important factors in the formation of HIV latent virus reservoir (Vandergeeten et al., 2012). For instance, IL-10, IL-8 and transforming growth factor –β (TGF-β) can produce long-term latent infectious cells by reducing T cell activation, which has been proved in *in vitro* experiments (Wilson and Brooks, 2011; Travis and Sheppard, 2014; Morris et al., 2017).

## The cell reservoir of HIV

Currently, CD4^+^T cells of resting memory constitute the major reservoir of latent HIV, which has widely been recognized by researchers (Campbell et al., 2018; Kwon et al., 2020; Moranguinho and Valente, 2020). However, a study reported that the rate of virus recurrence after the termination of ART therapy is much higher than the replication rate of CD4^+^T cells, indicating the presence of other reservoirs of latent HIV in addition to CD4^+^T cells (Chun et al., 2000). It has been proved that a variety of cells in circulating blood can also exist in the form of a latent reservoir of HIV after infection, which is associated with a variety of diseases and affect the development of HIV (Burdo, 2019; Kristoff et al., 2019; Veenhuis et al., 2019).

### CD4^+^T lymphocytes of resting memory

CD4^+^T cells of resting memory are stable reservoirs of latent HIV infection (García et al., 2018). A previous study stated that there was no significant loss of integrated HIV DNA in resting memory T cells over time, with a half-life of about 25 years (Murray et al., 2014). Resting memory T cells can be divided into different subtypes, including primitive T cells (T_N_) and memory T cells (T_M_) (Terahara et al., 2019). T_M_ cells are divided into central memory T cells (T_CM_), transitional memory T cells (T_TM_), effector memory T cells (T_EM_) and stem cell memory T cells (T_SCM_) (Corneau et al., 2017; Gálvez et al., 2021). Viral DNA has been detected in all of the above mentioned resting CD4^+^T cell subpopulations in HIV patients (Zerbato et al., 2016), suggesting that CD4^+^T lymphocytes of resting memory may be major hosts of latent viral infection.

Studies have confirmed that T_M_ is an important part of the reservoir of latent HIV. In terms of function, T_SCM_ cells exhibit higher response ability upon stimulation by homologous antigen (Lugli et al., 2013). Moreover, some T_SCM_ cells express CC chemokine receptor 5 (CCR5) and C-X-C chemokine receptor 4 (CXCR4), the main co-receptors for HIV entry, and making them susceptible to HIV infection (Tabler et al., 2014). In addition, T_SCM_ cells have a very long half-life and thus form the most stable part of the latent reservoir (Cartwright et al., 2016). T_SCM_ cells can produce highly differentiated cells, such as T_CM_ and T_EM_, while T_TM_ are intermediate phenotypes between T_CM_ and T_EM_ cells, each of which maintains its latent reservoir (Kulpa et al., 2019). As mentioned above, in addition to the virus exposure, there are many other factors influencing the latent infection of the resting CD4 + T cells, as some of the negative control cells activate the immune factors. Previous study reported that some factors in T_EM_, T_TM_, and T_CM_ are more active, which affect the reservoir of lurking in the formation these cells (Kwon et al., 2020).

Compared with T_M_, viral DNA could be detected in T_N_ cells despite the low frequency of HIV infection (Gibellini et al., 2017). At the same time, data have shown that infected T_N_ cells are treated with drugs that reverse latency. The number of extracellular virions produced by T_N_ in each infected cell was the same as that of T_M_ cells, suggesting that T_N_ cells with latent infection may also be the important source of the virus after treatment interruption or failure, that is, they are important hosts of latent HIV infection, and should not be ignored because of their low infection frequency (Zerbato et al., 2019).

### Mononuclear macrophages

In addition to resting memory CD4^+^T cells, myeloid cells, especially mononuclear macrophages, are currently considered to be important reservoirs of the latent HIV (Kumar et al., 2014). Mononuclear macrophages are considered to be early targets of HIV infection, because both CD4 receptors and CCR5 or CXCR4 co-receptors express on their surfaces (Lee et al., 1999). It has been shown that mononuclear macrophages play a key role in the innate immune response to pathogens, viral persistence, and viral library formation (Abreu et al., 2019; Kruize and Kootstra, 2019). Monocytes from bone marrow circulate in the blood and migrate to tissues to differentiate into various types of macrophages. Although monocytes rapidly differentiated into macrophages, studies have suggested that HIV has been detected in monocytes (Saini and Potash, 2014). In addition, macrophages have a long life span, found in almost all tissue in the body. Macrophages are relatively resistant to HIV-induced apoptosis, and can remain in antiviral treatment, so they are considered to play a key role in the establishment and persistence of latent HIV reservoir (Kruize and Kootstra, 2019). Brown et al. designed a long-term cultured *in vitro* model of macrophages infected with green fluorescent protein (GFP) labeled recombinant HIV. The results revealed that macrophages can establish incubation periods *in vitro* (Brown et al., 2006).

A quantitative virus outgrowth assay (QVOA) was used to measure the myeloid cells of latent infection in the simian immunodeficiency virus (SIV)-rhesus monkey model. The findings revealed that mononuclear macrophages with latent infection were detected in blood Broncho-alveolar lavage fluid, lung, spleen and brain (Abreu et al., 2019a; Abreu et al., 2019b), indicating that these cells persist during SIV infection and may act as latent viral reservoirs during antiretroviral therapy. Moreover, the isolated viruses produced by macrophages can infect activated CD4^+^T cells, suggesting that latent infected macrophages can re-infect after treatment interruption.

### Dendritic cells

Dendritic cells (DCs) are a heterogeneous group of antigen-presenting cells, playing an important role in immune response (Worbs et al., 2017; Balan et al., 2019; Yin et al., 2021). DCs are divided into myeloid dendritic cells (mDCs) and plasmacytoid dendritic cells (pDCs) based on maturity and origin (Verna et al., 2021). mDCs and pDCs, in an *in vitro* experiment were found to have different susceptibility to HIV (Smed-Sörensen et al., 2005; Groot et al., 2006).

Low levels of preHIV can be detected in DCs, suggesting that DCs may play a role in the HIV reservoir (Pope et al., 1995). Through the formation of infectious or virologic synapses (McDonald et al., 2003), DCs will transfer the infection to antigen-specific CD4^+^T cells upon HIV encounter (Loré et al., 2005), thereby weakening the establishment of anti-HIV immune response. In addition, HIV can appear in DCs and fuse with T cell membranes (Yu et al., 2008). Subsequently, DCs may be a potential target for HIV infection and latency due to surface pattern recognition receptor interactions with pathogens (Cardinaud et al., 2017).

Follicular dendritic cells (FDCs) present in secondary lymphoid tissue are the major sites of HIV infection during antiretroviral therapy (Olivetta et al., 2020; Ollerton et al., 2020). The virus is captured as an immune complex on its surface, the resulting complex is highly infectious to CD4^+^T cells (Keele et al., 2008). A previous study reported that FDCs in mice can retain the captured virus particles in an infected state for at least 9 months *in vivo* (Smith et al., 2001). Data also suggested that HIV captured by FDCs is capable of replication and exhibit greater genetic diversity than viruses found in other tissues or cells, and hence is an important host for an infectious and diverse group of HIV (Keele et al., 2008).

### Hemopoietic progenitor cells

Since hematopoietic progenitor cells (HPCs) express HIV receptors, long-term infection of HPCs may also be an important factor in the residual HIV after treatment (Carter et al., 2011). It has been confirmed that different subtypes of HIV can infect HPCs *in vivo* or *in vitro* (Li et al., 2017). To study latent infection in HPCs, Carter et al. (2010) conducted experiments using cells with different infection states. the findings revealed that viral gene expression was induced upon the treatment of latent HPCs with cytokines, stimulated the differentiation of bone marrow cell lines (granulocyte-macrophage colony-stimulating factor GM-CSF and tumor necrosis factor TNF-α), suggesting that HIV could infect HPCs and cause both active and latent infections.

Studies have shown that CD34+HPCs expressing CD4 CCR5 or CXCR4 and other receptors and co-receptors are associated with the susceptibility of these cells to HIV (Carter et al., 2010; McNamara et al., 2013). In addition, McNamara et al. (2013) assessed HIV infection of CD34+HPCs in 9 HIV patients who received ART and had no detectable viral load for at least 6 months. In four of the nine patients, preHIV genomes were detected in CD34 cells at a frequency of 3-40 genomes per 10,000 cells, suggesting that HPCs can serve as a reservoir of latent HIV.

### Astrocyte

HIV can invade the central nervous system (CNS), causing neuroinflammatory immune activation and neurodegenerative alterations, resulting in HIV-related neurocognitive impairment (Knight et al., 2018; Knight et al., 2020). Astrocytes are the most abundant cell type in CNS and play a vital role in maintaining the CNS homeostasis and regulating blood flow in response to injury and diseases (Guttenplan and Liddelow, 2019).

Due to the absence of CD4 receptors, astrocytes lead to restricted HIV infection. Although the proportion of HIV binding to astrocytes is low, HIV DNA and RNA have been found in astrocytes using *in-situ* hybridization laser capture anatomy and nested polymerase chain reaction (PCR) (Churchill et al., 2006; Chong et al., 2018), suggesting that astrocytes can be infected by HIV. In addition, studies using human astrocytes and human peripheral blood mononuclear cell chimeric model further confirmed that astrocytes are the host of HIV (Lutgen et al., 2020).

HIV infection is found to be acquired through pH-dependent endocytosis, which consumes most of the virus particles, however, pH-dependent endocytosis still may be an important pathway for HIV to establish incubation in astrocytes (Chauhan and Khandkar, 2015). Previous studies reported that Tat protein expression can affect HIV infection and latency establishment in astrocytes. Tat protein promotes the formation of HIV latency by inducing tri-methylation of histone H3 on Lys27 (H3K27me3) expression in astrocytes. A decrease in Tat protein expression decreases the formation of latent HIV infected cells (Chauhan and Khandkar, 2015). Meanwhile, the *in vitro* use of latency reversing agents (LRAs) has further identified astrocytes as part of the reservoir of latent HIV (Schneider et al., 2015).

### Other cells

In addition to the aforementioned cells, several other cells types are also reported to form latent reservoirs of HIV. Macrophages and microglia in the central nervous system are the main antigen-presenting cells that can be infected with HIV. Previously, researchers detected HIV DNA in these pair of cell isolated from the brain tissue of five dead individuals, proving them as the main cellular hosts for latent HIV (Thompson et al., 2011). At the same time, microglia can be sustained for a long time and can proliferate *in situ*, and hence proved to be a key drive for viral reservoirs (Wallet et al., 2019).

Epithelial cells are also susceptible to HIV. Renal tubular epithelial cell co-cultured with infected T cells becomes susceptible to HIV. HIV DNA and RNA were detected in renal tubular epithelial cells by *in situ* hybridization of biopsies collected from patients with HIV-associated nephropathy (Katuri et al., 2019). In addition, it has been reported that integrated HIV DNA can be detected after *in vitro* infection of liver cell lines and primary liver cells, and the release of infectious viruses was also found in the liver epithelium (Ganesan et al., 2018).

## Tissue reservoir of HIV

Studying the latent HIV reservoir in tissues is challenging due to the difficulty of tissue sampling. In recent years, the research on the latent HIV reservoir in tissue has mainly focused on the autopsy of non-humans primates and humans (Cohn et al., 2020). Lymph nodes (LN) and gut-associated lymph tissue (GALT) were found to be the major tissue reservoirs rich in viruses and with high frequency of infected cells (Churchill et al., 2016). In the course of HIV infection, B cell follicles in the lymphoid structure actively reject effector CD8^+^T cells to maintain normal B cell function, thereby providing favorable conditions for the formation of latent HIV reservoir (Connick et al., 2014; Fukazawa et al., 2015). Previous studies suggested that the gut may be the body’s largest reservoir of HIV. In addition, by examining the vaults of SIV-infected rhesus monkeys, the researchers found that the vast majority (>98%) of storage stocks are present in the gut (Estes et al., 2017).

Previously, a human autopsy study revealed that the HIV provirus was detected in 28 tissues, including the liver, spleen, genital tract and brain (Chaillon et al., 2020). Several major HIV reservoir cells, such as resting memory CD4^+^T cells, dendritic cells, macrophages and microglia, are widely located in these tissues (Honeycutt et al., 2017; Wallet et al., 2019). Some of the studied sites are termed sanctuary sites, which are protected from ART penetration (the brain and testis) and pose additional challenges for HIV treatments (Fletcher et al., 2014).

**Figure 2:**
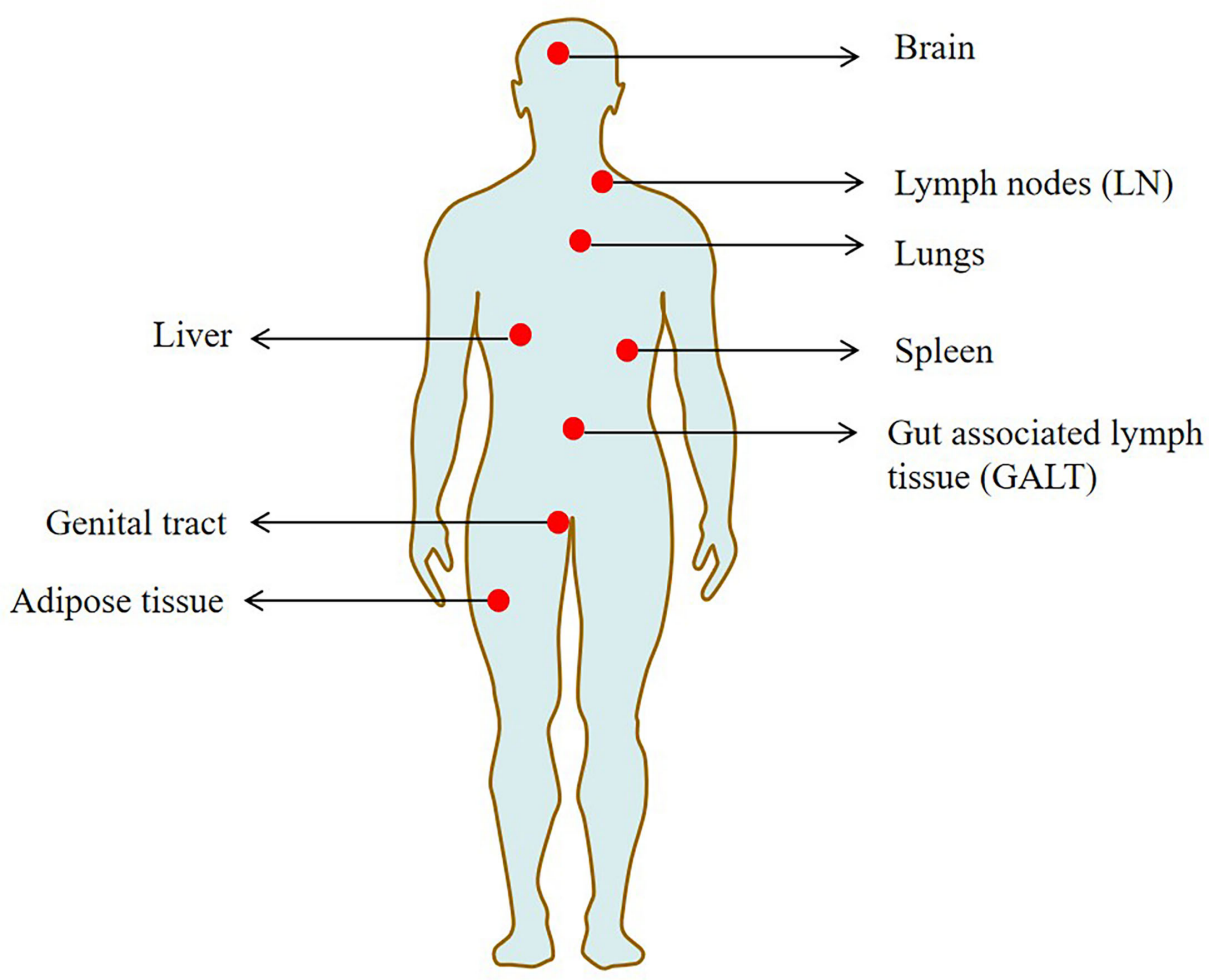
The main tissue locations of latent HIV reservoir.

Tissue macrophages such as those found in seminal vesicles, urethra, adipose tissue and liver tissue are considered to be important hosts of HIV, including macrophages (Deleage et al., 2011; Ganor et al., 2013; Damouche et al., 2015). In addition, infected macrophages have been found at low but detectable frequencies in lung and duodenal tissue of patients on ART with undetectable plasma viruses (Cribbs et al., 2015). The reproductive tract is also rich in macrophages and may be an important reservoir of latent HIV, where antiretroviral drugs are difficult to enter. Study has found that the male reproductive tract may also exist in the latent HIV (Ganor et al., 2019). Additionally, tissue resident memory CD4^+^ T cells in the female genital tract (particularly the cervix) are highly enriched with HIV DNA (Cantero-Pérez et al., 2019). **Figure 2** shows the main tissue reservoirs of HIV.

## Detection of latent HIV reservoirs

At present, detection methods for finding accurate, sensitive, and scalable reservoirs of latent HIV are important in the treatment of reservoirs. Virus outgrowth assay (VOA) is considered the gold standard for the quantification of latent HIV reservoir. It measures the replicability of the original virus by diluting resting CD4^+^T cells and activating the intracellular viral gene expression, resulting in inducing the release of HIV from latent infected cells (Wang et al., 2018). However, this method is time-consuming, expensive, and requires a large amount of blood culture. Moreover, single stimulation is not sufficient to activate all latent viruses, which underestimates the size of latent HIV reservoir (Massanella et al., 2018; Wonderlich et al., 2019). Detection of HIV DNA based on PCR is relatively simple and rapid method for the detection of the latent reservoir, and provides a supplement for the VOA test (Ho et al., 2013). Quantitative real-time polymerase chain reaction (qPCR) for detection of HIV DNA to determine the amount of HIV DNA carried (Thomas et al., 2019), digital PCR (dPCR) for detection of HIV DNA for absolute quantitative HIV (Henrich et al., 2017), and Alu-polymerase chain reaction (Alu-PCR) for detection of HIV integration the amount of DNA is used to evaluate the size of the latent reservoir of HIV (Lada et al., 2018). PCR has made rapid development in the determination of latent reservoir of HIV, however, the size of the reservoir may be overestimated because PCR cannot distinguish intact from defective original virus (Ho et al., 2013).

To counter the above mentioned challenges, several innovative approaches have been developed for detecting latent HIV reservoirs including intact proviral DNA assay (IPDA), which differentiates intact and defective viruses, and screen different defective viruses by plasmid control (Gaebler et al., 2021). It was found that 90% of the defects in the viruses occurred in the encapsulation signal (ψ) and *env* regions. With the detection of ψ and env regions by dPCR, complete viruses and defective viruses could be identified (Bruner et al., 2019). Tat/Rev induced restricted dilution test (TILDA) is used to measure the frequency of CD4^+^T cells of latent HIV proto-virus. Researchers discovered that changing the preamplification settings did not affect on the Tat/Rev multiple splicing RNA assay, confirming the stability of the assay and supporting its adaptability to limited modifications to ensure better clinical use in latent HIV reservoirs (Bertoldi et al., 2020; Lungu et al., 2020). ISH and flow cytometry, have a higher sensitivity to replication of the original virus, and can phenotype host cells (Deleage et al., 2018; Pardons et al., 2019). There was a study that described how to use this technology to address some of the major questions remaining in the HIV feld in the era of ART.They discussed how CD4^+^T cell responses to HIV antigens, both following vaccination and HIV infection, can be characterized by measurement of cytokine mRNAs. They also described how their development of a dual HIV mRNA/protein assay (HIV^RNA/Gag^ assay) enables high sensitivity detection of very rare HIV-infected cells and aids investigations into the translation competent latent reservoir in the context of HIV cure (Baxter et al., 2017). The emergence and development of these detection techniques provide strong evidence for the study of latent HIV reservoir. **Table 1** summarizes the testing methods for latent HIV reservoirs.

**Table 1:** The different detection methods of latent HIV reservoir.

	Assay	Advantages and disadvantages	References
Viral outgrowth assay (VOA)	The replicable proto-virus was measured by diluting resting CD4^+^T cells to activate intracellular viral gene expression and induce the release of HIV from latent infected cells	The gold standard for measuring latent HIV reservoirs But it is expensive, time-consuming, requires a lot of blood culture and underestimates the size of the latent reservoir	(Wonderlich et al., 2019) (Massanella et al., 2018) (Fun et al., 2017) (Badia et al., 2018)
PCR	Detection of HIV DNA using primers/probes	Simple, fast, and provides a supplement for VOA experiment But it’s possible to overestimate the size of the repository	(Ho et al., 2013)
qPCR	Testing for HIV DNA to determine the amount of HIV DNA carried		(Thomas et al., 2019) (Vandergeeten et al., 2014)
ddPCR	Detection of HIV DNA for absolute HIVquantification		(Henrich et al., 2017) (Lada et al., 2018)
Alu-PCR	The amount of integrated HIV DNA was measured to evaluate the size of the latent reservoir		(Vandergeeten et al., 2014) (Lada et al., 2018)
Intact proviral DNA assay (IPDA)	Complete proviruses were measured by multiplex digital PCR, and different defective proviruses were verified by plasmid control	Fast and able to distinguish between intact and defective proviruses But it didn’t screen the whole genome	(Bruner et al., 2019)
TILDA	CD4^+^T cell frequency of latent HIV proto-virus was measured by Tat/Rev induced restricted dilution test	High sensitivity and stability But the transcripts measured may come from defective provirus genomes	(Bertoldi et al., 2020) (Frank et al., 2019)
ISH and flow cytometry	mRNA and viral proteins were measured after T cell activation	It has high sensitivity and can phenotype host cells But it can not prove that RNA or proteins are made by replicating protoviruses	(Baxter et al., 2017) (Deleage et al., 2018) (Pardons et al., 2019)

## Strategies for removing latent HIV reservoirs

### Shock and kill strategies

Shock and kill therapy use drugs to activate the gene transcription (shock) of HIV lurking in cells, and then kill the virus (kill) through the body’s immune system, ART or other intervention methods to eliminate the latent HIV. It is one of the most effective methods to remove the latent HIV (Sadowski and Hashemi, 2019; Nixon et al., 2020). LRAs are used for activation of viral transcription, the production of viral proteins, release of virus particles, and, effectively eliminating latent HIV in combination with ART (Spivak and Planelles, 2018; Delannoy et al., 2019). Therefore, LRAs play an important role in this process. It has been found that many substances can be used as LRAs to activate latent HIV reservoir, such as: 1) cytokines or receptor agonists, such as IL2, IL7, IL15, toll-like receptor 2,3(TLR2, 3) agonists, etc. The drugs were found to reactivate the expression of HIV, but they did not eliminate the latent infection cells, or affect the size of the HIV reservoir (Rochat et al., 2017; Madrid-Elena et al., 2018). 2) Epigenetic modification enzyme inhibitors, such as histone deacetylase inhibitors (HDACi) histone methyltransferase inhibitors (HMTi), and DNA methylation inhibitors (DNMTi), etc. HDACi and HMTi have been shown to reactivate HIV expression to some extent *in vitro* and *in vivo* (Archin et al., 2017). In addition, DNA methylation inhibitor 5-AzadC can also induce HIV expression *in vitro*, while its oxidation analog 5-AzaC is unable to do so (Bouchat et al., 2016). 3) Cellular signaling modulators, such as protein PKC receptor agonist prostratin and bryostatin, activate the protein kinase C (PKC) pathway to release NF-κB and positive transcription elongation factor b (pTEFb) from inactive complexes and increase pTEFb expression, ultimately leading to HIV reactivation (Wang et al., 2017; Schwartz et al., 2017). In addition, antioxidants, AKT regulators, protein phosphatase 1, and many other substances induce the expression of HIV proto-virus (Chirullo et al., 2013; Doyon et al., 2014; Smith et al., 2015; Tyagi et al., 2015), however, their specific mechanism of action remains unclear and needs further study. **Table 2** summarizes commonly used latent reversal agents.

**Table 2:** The commonly used latent reversal agents.

Category	Examples	Mechanism of action	References
Cytokines or receptor agonists	IL2、IL7、IL15、TLR2,3	Reactivation of HIV expression	(Rochat et al., 2017) (Rasmussen et al., 2016)
Epigenetic modification enzyme inhibitors	HDACi、HMTi、DNMTi	Reactivation of HIV expression *in vitro* and *in vivo*	(Archin et al., 2017) (Bernhard et al., 2011)
Cell signal regulator	Prostra、BryostatinC	Activation of PKC pathway results in the release of NF-κB and pTEFb from inactive complex and increases pTEFb expression	(Wang et al., 2017) (Schwartz et al., 2017)
Others			
Antioxidant	Auranofn (AF)	The mechanism of action is still unclear	(Chirullo et al., 2013)
AKT regulator	Disulfram		(Doyon et al., 2014)
Protein phosphatase 1	SMAPP1		(Smith et al., 2015) (Tyagi et al., 2015)

Schwartz et al (Schwartz et al., 2017) investigated the reactivation potential of compounds releasing active pTEFb in combination with PKC agonists. The combination of HMBA/BETi and PKC agonists led to strong synergistic activation of HIV expression in several *in vitro* post-integrated latency cell line models (Darcis et al., 2015). Continuous treatment with demethylation agents (5-AZADC) and clinically tolerated HDACi was also shown to be more effective than corresponding concurrent treatment in inducing HIV gene expression *in vitro* and *ex vivo (* Bouchat et al., 2016). These datas demonstrate the importance of treatment schedules in combination with LRAs for HIV activation.

The ability of LRAs to activate *in vitro* is correlated with the size of the HIV reservoir.However, some patients have very low or extremely high reactivation relative to the size of their reservoir (Darcis et al., 2017). Timely administration of LRAs in reactivation trials and a better understanding of the variability of reactivation in patients are important.A defective Cas9 (dCas9) protein fused to activators may be a new tool to reactivate potentially infected cells. CRISPR/dCas9 may be used to reactivate latent HIV *in vitro* experiments (Zhang et al., 2015). Similarly, CRISPR/dCas9 synergistically activates HIV when used in combination with HDAC inhibitors and PKC activators (Limsirichai et al., 2016).

Exosomes, as a way of material and information transmission between cells, are the key features in viral infection (Chen et al., 2021). Research studies on the activation of latent HIV reservoir have found exosomes to induce the activation of resting CD4^+^T cells infected with HIV through different mechanisms. For example, exosomes from HIV-infected cells have been shown to activate resting CD4^+^T lymphocytes *via* ADAM17 and tumor necrosis factor-α (TNF-α) dependent mechanisms (Arenaccio et al., 2014; Arenaccio et al., 2015). In addition, HIV-coded Tat protein is an effective viral transcription transactivator. Tat protein is encapsulated in exosomes for targeted delivery to latently infected CD4^+^T lymphocytes, which reactivate the virus. When combined with an HIV LRA, the expression of HIV mRNA increases by >30-fold (Tang et al., 2018).

Despite the reactivation of latent HIV, LRAs alone cannot significantly reduce the size of the reservoir, suggesting that kill is needed to destroy HIV-infected cells after shock therapy (Castro-Gonzalez et al., 2018; Sadowski and Hashemi, 2019; Maina et al., 2021). ART is an important method used to reduce the activated virus, and HIV-specific CD8+T cells play a key role in eradicating HIV hosts. However, the immune system of ART patients is unable to produce sufficient anti-HIV cytotoxic CD8+T cell response, therefore, it is unable to eliminate the activated cells in large numbers. Consequently, it is necessary to enhance cellular or humoral mediated immune response to promote cell apoptosis, and eliminate the latent HIV (Kim et al., 2018).

### Block and lock

Contrary to the shock and kill strategy, the block and lock strategy permanently silence the original HIV to prevent the virus from rebounding. The block and lock strategy prevents the transcription and reactivation of HIV in the latent infection cells to inhibit the emergence of the latent virus (Moranguinho and Valente, 2020; Vansant et al., 2020). HIV transcription is a complex process involving many virus proteins and cytokines, including Tat proteins to induce viral transcription extension, host transcription factors, transcription suppressors, etc. Alterations in any of these factors may silence HIV transcription (Vansant et al., 2020). In addition, chromatin and epigenetic landscape and HIV integration sites also play important roles in viral transcription (Pearson et al., 2008; Tyagi et al., 2010).

To address these potential targets affecting latent HIV transcription and silencing, several block and lock strategies have been proposed: 1) Inhibition of Tat protein expression through didehydro-cortistatin A (dCA). dCA is an effective inhibitor of Tat protein, which can block the transcription and reactivation of HIV by LRAs in CD4^+^T cells. It has been found that dCA treatment can reduce or delay virus recurrence. Meanwhile, dCA has a strong specificity for Tat protein and plays an important role in inhibiting virus reactivation (Kessing et al., 2017). 2) Inhibition of HIV integration and relocalization of the residual original virus by LEDGINs. Lens epithelium-derived growth factor p75 (LEDGF/p75) is a determinant of HIV integration site selection and affects the transcription status of the original virus (Debyser et al., 2018). LEDGINs are small molecular inhibitors of the interaction between LEDGF/p75 and integrase (Christ et al., 2010), which can impede HIV integration and the catalytic activity of HIV integrase (IN), to increase the proportion of protovirus with transcriptional silencing phenotype (Symons et al., 2018). 3)Small interfering RNA (siRNA) is used to maintain the repressive heterochromatic landscape at the HIV5’ LTR promoter (Ahlenstiel et al., 2015). siRNA silenced HIV genes transcription by recruiting Argonaute1(AGO1), histone deacetylase 1, and histone methyltransferase (Méndez et al., 2018). 4) Promoting chromatin transcription complex (FACT) is also one of the important regulatory factors of HIV transcription. Curaxins CBL0100, an anticancer drug, was discovered to suppress HIV replication and reactivation by inhibiting RNAPII-mediated transcriptional elongation in a Tat-dependent manner (Jean et al., 2017). 5) Mammalian target of rapamycin (mTOR) inhibitors, hinders HIV reactivation by down-regulating cyclin-dependent kinase 9 (CDK9) phosphorylation and blocking NF-κB signal transduction (Besnard et al., 2016).

**Figure 3:**
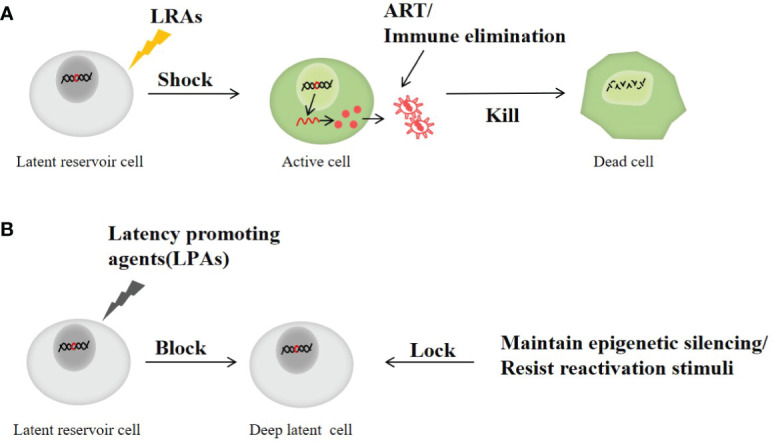
The main process of shock and kill and block and Lock therapy to clear latent reservoir of HIV. **(A)** Shock and kill strategy: the latent HIV is activated with a LRAs and then killed by ART or immunotherapy. **(B)** Block and lock strategy: by preventing the transcription and reactivation of HIV in latent infection cells to inhibit the emergence of latent virus and prevent virus rebound.

In addition, it has been found that heat shock protein 90(HSP90) inhibitors, Jak (Janus kinase)-STAT inhibitors, kinase inhibitors and bromodomain protein 4(BRD4) regulators also play corresponding roles in inhibiting the latent HIV transcription and reactivation (Vansant et al., 2020). Although many compounds are still in the early stages of HIV research, their emergence offers new possibilities for cure. **Figure 3** shows the main process of shock and kill, and block and lock to eliminate the latent reservoir of HIV.

### Immunization therapy

When the latent HIV is activated by LRAs, immunotherapy can kill or eliminate HIV-infected cells. Moreover, immunotherapy can stimulate the natural immunity of HIV-infected cells (Ferrari et al., 2017). The immune response can be enhanced by regulating cytokines and interleukins (Pankrac et al., 2017). IL-15 enhances the activity of natural killer cells (NK), thereby enhancing cell elimination after LRAs treatment (Garrido et al., 2018). Subsequently, a polo-like kinase 1 (PLK1) inhibitor can also enhance the anti-HIV effect of DCs (Gringhuis et al., 2017).

Broadly neutralizing antibodies (bNAbs) target specific proteins outside the HIV, thereby reducing infectivity. bNAbs can significantly reduce viral load (Parsons et al., 2018). In addition, bNAbs can also be used in combination with shock and kill therapy to eliminate infected cells. When LRAs activate potentially infected cells, bNAbs eliminate them (Martinez-Navio et al., 2019). Antibody therapy can also promote apoptosis of activated cells by targeting surface markers. In addition, the conjugation of antibodies with cytotoxic compounds or drugs can be used to target and kill specific cell types recognized by antibodies. Antibodies used in this class of antibody drug conjugate (ADC), including bNAbs, recognize HIV gag or HIV *env* gene products and, may provide effective killing of latent infected cells (Dan et al., 2018).

At the same time, the CD8^+^T cells isolated from HIV patients can modify their specificity *in vitro* to produce anti-HIV cytotoxic T lymphocytes (CTL) response and are amplified and reintroduced into patients, which can also promote their immune response to the reactivated cells, to eliminate the latent infection cells (Yang et al., 2016).

In addition to the above strategies, some other methods against latent HIV reservoir have been proposed, such as interferon (IFN) combined with ART therapy, stem cell transplantation (Kuritzkes, 2016), gene editing including CRISPR (Das et al., 2019; Herrera-Carrillo et al., 2020; Atkins et al., 2021), zinc-finger nucleases (Wayengera, 2011; Ahlenstiel et al., 2020) etc. Target cells can be induced to develop HIV resistance through gene editing, such as gene editing to remove the CCR5 gene, and reduce the binding of HIV to CD4 cells (Yang et al., 2016; Schwartz et al., 2017). Each method has its own characteristics and plays a certain role in potentially controlling the latent virus reservoir.

## Discussion

The establishment of a latent HIV reservoir is a complex process, and can exist in a variety of cells and tissues throughout an infected person’s body, making it difficult to detect and eliminate. With the deepening of HIV research, the existence of latent HIV reservoir has gained extensive attention. Subsequently, great progress has been made in the research of latent HIV reservoir. Many methods for the detection and elimination of latent HIV reservoir have been proposed.

At present, activating latent HIV to kill, is still the key to eliminate the reservoir of latent HIV. Therefore, people are committed to finding safe and efficient activators to activate the latent HIV. Recently, exosomes have gradually become the focus of HIV clinical research. Previous studies have found that exosomes can activate latent HIV reservoir cells through various mechanisms, and various substances carried by exosomes play an important role in the occurrence and development of HIV, which has broad application prospects in the clearance of latent HIV reservoir and the treatment of HIV (Barclay et al., 2017; Chandra et al., 2021). In addition, clinical research proved that traditional Chinese medicine plays an irreplaceable role in the treatment of HIV (Li et al., 2020; Qian et al., 2021). Our study found that traditional Chinese medicine (TCM) and its related components can also activate the latent HIV, providing a new direction for TCM treatment of HIV. At the same time, new mechanisms for activating latent HIV reservoir have been proposed, which provide the possibility for the elimination of latent reservoir.

However, research on latent HIV reservoir is still in the initial stage, and further exploration is needed to better understand the intricacies of possible mechanism, location, detection, and clearance methods, to achieve a complete elimination of latent HIV reservoirs.

## Author contributions

JC, TZ and YZ conceived of the presented idea. SL, HC and DC researched on the background of the study. CL and WL critically reviewed the manuscript. All authors contributed to and approved the final manuscript.

## Funding

This research was funded by the National Natural Science Foundation of China (81603552), the Natural Science Foundation of Beijing (7212172), the Pilot project of public welfare development and reform of Beijing Municipal Medical Research Institutes (2019-6) and the Special project for the construction of high-level health technical personnel in Beijing Health System (2022-2-024).

## Acknowledgments

The authors would like to thank all the investigators and the staff of the Second People’s Hospital in Lianyungang City, China, for the provision of clinical isolates.

## Conflict of interest

The authors declare that the research was conducted in the absence of any commercial or financial relationships that could be construed as a potential conflict of interest.

## Publisher’s note

All claims expressed in this article are solely those of the authors and do not necessarily represent those of their affiliated organizations, or those of the publisher, the editors and the reviewers. Any product that may be evaluated in this article, or claim that may be made by its manufacturer, is not guaranteed or endorsed by the publisher.
